# The transition to clinical expert: enhanced decision making for children aged less than 5 years attending the paediatric ED with acute respiratory conditions

**DOI:** 10.1136/emermed-2015-205211

**Published:** 2016-08-05

**Authors:** Leah Bowen, Alison Shaw, Mark D Lyttle, Sarah Purdy

**Affiliations:** 1 Centre for Academic Primary Care, University of Bristol, Bristol, UK; 2 Emergency Department, Bristol Royal Hospital for Children, Bristol, UK; 3 Faculty of Health and Applied Sciences, University of the West of England, Bristol, UK

**Keywords:** paediatric emergency med, emergency care systems, primary care

## Abstract

**Background:**

Rates of unplanned paediatric admissions are persistently high. Many admissions are short-stay events, lasting less than 48 hours.

**Objective:**

This qualitative research explores factors that influence clinical decision making in the paediatric ED (PED) for children under 5 attending with acute respiratory conditions, focusing on how management decisions adapt with increasing experience.

**Method:**

Semi-structured interviews were conducted with 15 PED clinicians (doctors, emergency nurse practitioners and registered nurses) with varying levels of experience in paediatric emergency medicine (PEM), emergency medicine or paediatrics. Audio-recorded interviews were transcribed and analysed thematically.

**Results:**

There were clear differences in decision-making approaches between experienced clinicians and junior staff. The latter were more risk adverse, relying heavily on guidelines, set admission criteria, clinical theory and second opinions. This was particularly true for doctors. ‘Informal’ learning was apparent in accounts from less-experienced doctors and nurses, whereby tacit knowledge and risk management played an increasing role in the development of clinical intuition that permitted rapid assessment and treatment of young patients.

**Conclusions:**

The emergence of intuition entwined with approaches to risk management and the role of these skills in clinical decision making, carry implications for the development of training programmes for clinicians working in PEM. Enhanced training for such groups to permit development of the supplementary skills described in this study could have the ability to improve care delivery and even reduce paediatric admissions.

**Key messages**

**What is already known on this subject?**

- Rates of paediatric emergency admissions, especially short-stay admissions for respiratory conditions, are persistently high.
- Many such admissions are for respiratory illnesses that may be amenable to primary care management.
- Decision making in the paediatric ED (PED) for children attending with episodes of acute respiratory illness remains unexplored. Greater understanding of issues from urgent care settings could provide a platform for successful management of many conditions in the community.

**What might this study add?**

- Clinicians working in the PED use a combination of clinical rules, supplemented by additional skills of observation, risk management and intuition to achieve clinical decisions in cases involving acute respiratory illness in children younger than 5.
- The supplementary skills of observation, risk management and intuition develop over the course of training and are used to good effect by experienced clinicians to arrive at rapid treatment decisions.
- Efforts should be directed towards training healthcare professionals in other settings to develop the skills identified to support management of children outside the ED.

## Introduction

Demand on NHS urgent care is stretched, with a significant number of trusts across the country failing to meet national care targets,^1^ ^2^ and unplanned hospital admissions in England and Scotland have been rising annually with the largest increases observed in the youngest children.^3–5^ Many hospitalisations are short in duration, lasting less than 48 hours, and more than half of all admissions are for potential primary care-sensitive conditions (PCSCs).^5^

Quantitative research has highlighted the issue of persistently high paediatric emergency admission rates, yet many complex factors behind these admissions are not understood. Parental perception (including concerns and preference for urgent care^6^ ^7^) coupled with the lack of availability of primary care services^8^ shape the way in which emergency care is used, but less is known about clinicians' propensity to admit or the factors that influence their decision making in this regard. Accounts from clinicians discussing paediatric admissions for respiratory illness are surprisingly few, given the growing demand by this population. In prior quantitative studies in this field,^8^ ^9^ questionnaires on clinical decision rules were unable to account for external and complex contextual factors that may impact decision making beyond biomedical indicators alone.

We therefore explored the decision-making process in clinicians of varying specialty and experience working in a paediatric ED (PED), relating to admission and discharge decisions while not excluding other areas of decision making. We focused on young children attending the PED, using respiratory illness as an index condition, and aimed to elucidate potentially modifiable clinician behaviours that could add to the understanding of the complexity behind rising short-stay admissions.

## Methods

### Study setting

Semi-structured interviews were conducted with clinicians in a tertiary children's hospital with a stand-alone PED, which delivers secondary care to local children and tertiary care to the South West of England. It sees 35 000 children per year and has an embedded eight-bed observation unit, which is managed and staffed by PED medical and nursing staff, functioning as a type C unit.^10^ Clinicians in this department comprise paediatric emergency medicine (PEM) consultants, general practice, emergency medicine and paediatric trainees (many of whom subspecialise in PEM) and emergency nurse practitioners (ENPs).

### Participant selection

Ethical approval for the study was granted by Faculty of Medicine and Dentistry Committee for Ethics, University of Bristol. Interviews were conducted between November 2013 and April 2014. Several sampling strategies were employed to maximise recruitment and participation, including the involvement of a gatekeeper, chain sampling, opportunistic and theoretical sampling.^11–13^

In the first instance, participation was open to all PED staff and was facilitated via a gatekeeper^13^ who enabled access to the research setting and clinicians.

Theoretical sampling^14^ was used towards the end of the study to purposively target participants of particular levels of experience to further examine issues relating to decision making that were emerging from earlier data.

### Data collection

Prior to interview, participants were provided with information explaining the purpose of the study, and written informed consent was obtained. Participants were asked to reflect on up to two instances when they were responsible for the care of a child under 5 years in the PED with a minor respiratory illness. These reflective cases facilitated discussion around the decision-making process and treatment approach taken. A definition of ‘minor’ respiratory illness was not provided; instead, it was the clinician's perception of ‘minor’ that was used as a basis for discussion.

Respiratory illnesses were used as representative of medical conditions likely to present to the PED and possibly result in hospitalisation.^15^ ^16^ Children aged under 5 years were selected as the age group at greater risk of admission, accounting for 68% of all paediatric emergency admissions in England in 2010.^3^

A topic guide was developed following pilot interviews with clinicians. Divergent accounts, including minor or more major variations in opinion, were identified and examined during discussions with individuals and throughout subsequent interviews in a process of constant comparison.^14^ ^17^ Interviews were audio-recorded and transcribed verbatim, each lasting 30–60 min.[Supplementary-material SM1]

10.1136/emermed-2015-205211.supp1 Supplementary data

### Data analysis

An interpretivist stance was adopted and applied throughout the design and conduct of the study and analysis of interview material. This approach is concerned with how the world is understood, experienced and interpreted by those who experience it.^18^ From this perspective, we sought to understand clinicians' beliefs on decision making, their rationales for the choices they make and the meanings they attach to their experiences.

Data were collected until theme saturation was achieved for this particular setting. Analysis was conducted alongside data collection to ensure that early findings fed into subsequent discussions which made identification of saturation apparent. By interview 10, the principle themes had been identified and were tested in subsequent discussions (interviews 10–15). In later interviews, new themes were not identified but instead, additional content emerged from interview accounts that aided the shaping and organisation of themes and subthemes.

Interview transcripts were analysed thematically using the constant comparative technique where data within and across each of the transcripts were repeatedly compared with to generate themes. This strategy served to identify divergent accounts and ensure the integrity of the findings.^14^ ^17^ Data analysis was supported by Nvivo 9, which was used to code, identify and organise themes and subthemes.

Open coding of transcripts was conducted by the first author (LB) generating an initial coding framework. Content was built into themes with subthemes through comparison across transcripts and supported by attention to divergent cases.^14^ ^17^

Each interview transcript was reviewed by SP and AS independently to enhance the trustworthiness of the analysis and build in alternative perspectives. The evolving coding framework was discussed regularly by the group to agree the major themes and produce appropriate thematic arrangement of interview content.

Interviewees were offered copies of their interview transcript to review and amend any content as they believed necessary. Just one participant accepted the transcript and no comments were received. Contact has been maintained with the host department via the gatekeeper who has commented on the themes presented, ensuring the trustworthiness and credibility of findings.

## Results

Characteristics of participants are provided in table 1. Twelve themes emerged from the interviews including themes relating to perceived appropriateness of attendances and admissions, clinicians' observation of parental factors that influenced direct attendance to the ED and primary care factors that contributed to ED demand. This paper will focus on a novel aspect of the research, concerning clinicians' decision-making approaches. Other themes will be reported in future articles.

**Table 1:** Participant profile by grade/band

Clinician (n)	Participant number	Years of experience (post-university qualification)
PEM consultants (2)	001	10+ years
	010	10+ years
PEM clinical trainee (1)	003	10+ years
GP trainees (2)	004	<5 years
	007	<5 years
Paediatric clinical trainee (1)	014	5–10 years
Emergency medicine trainees (2)	006	5–10 years
	008	<5 years
Emergency nurse practitioner—band 7/6 (1)	011	10+ years
Emergency nurse practitioner—band 7 (1)	013	10+ years
Senior staff nurse—band 6 (2)	015	10+ years
	012	5–10 years
Staff nurse—band 5 (1)	009	5–10 years
Research/staff nurse (50:50—band 6/5) (2)	002	<5 years
	005	<5 years
Total participants=15		

GP, general practice; PEM, paediatric emergency medicine.

The results presented here relate to 3 of the 12 major themes emerging from the study that are concerned with clinicians' decision-making approaches. The first theme of *Perception of factors influencing decision making* includes subthemes of perception of minor respiratory illness, admission/discharge options and risk management.

The second theme, *Assessment of severity* presents the factors which were essential in informing the clinician's approach in the individual patient cases selected for discussion. This theme encompasses subthemes covering clinical observation to support decision making.

The final theme of *Transition to expert* develops theory relating to how staff in the PED acquired decision-making skill as a result of experience gained in the setting. Here, four subthemes are presented to illustrate the learning journey.

### Perception of factors influencing decision making

Participants were asked to consider their perception of ‘minor respiratory illnesses’. Most commonly, mild presentations of bronchiolitis in infants, viral-induced wheeze and croup in older children were discussed as typical respiratory presentations to the PED.

Doctors tended to discuss decision making for this group of patients in relation to admission/discharge options, while nursing staff drew on their experiences of patient observation typically during triage (all nursing staff in the department were triage trained).

Managing risk was central in discussions about clinical decision making. In this context, risk was described by participants as balancing the safety of the patient (typically ensured by admission) against the possible hazards, associated with discharge and deterioration. Clinicians further described that it was not always possible to predict which children with minor respiratory illness would become seriously unwell and require escalating medical or nursing intervention. When deciding whether to discharge patients with self-care advice only, or provide therapy, assessment of illness severity ranging from mild to life-threatening presented a significant challenge. This decision was often complicated by further uncertainty regarding the child's likely response to treatment, age and perceived vulnerability.

‘I mean bronchiolitis is serious, or it is a scale I guess. So you can have the very well babies who are just a little bit snotty, but completely well with it, to other ones in intensive care ventilated’.

[Research/Staff Nurse 002]

‘You just don't know [sometimes] which way they'll go. They [children] deteriorate quickly but bounce back quickly too. It's always best to keep an eye on them’.

[Clinical Trainee Emergency Medicine 006]

### Assessment of severity

To identify children who required immediate intervention or escalation of care, clinicians described three skills to supplement physiological parameters in assessing illness severity:

- Observation of patients' clinical signs (including appearance).
- Observation of patients' behaviour.
- Intuition.

Observation of children's clinical signs consisted of clinical assessment and identification of ‘text book’ signs of respiratory distress such as ‘tracheal tug’ and sounds audible with a stethoscope. According to participants, these features may indicate severe illness; attention to these signs is consistent with analytical decision-making processes in which evidence is gathered to influence a decision. In the quotes below, two clinicians describe clues that indicated a child was more severely unwell. These were based either on taught signs (first participant) or on a mix of text book signs and personal experience (second participant).

‘Whether they're pale or becoming ashen coloured. Whether they're mottled’.

[Clinical Trainee GP 004]

‘They are sucking in at the ribs and you can see that they are working really hard at breathing. They are overcompensating. They are using all their accessory muscles to breathe’.

[Research/Staff Nurse 005]

Behavioural clues in the child provided further evidence of illness severity. Participants mentioned observation of age-appropriate interaction with caregivers and clinicians. One behavioural sign, ‘playing’, was suggested to be highly significant and a feature absent in many seriously unwell children. Behavioural clues were described as significant by five paediatric doctors and nurses, and two non-paediatric doctors.

‘Do I really think that a sick child is going to be playing? No, to be honest. A child with Pneumonia certainly isn't playing’.

[Clinical Trainee Paediatrics 014]

‘So they’re [the child] running around, and they’re carrying out, for them, what is their normal activities, and responds well to Salbutamol, and carries on playing’.

[Nurse Band 6 011]

The unwillingness of children to interact normally was important even if clinical observations were satisfactory. These cases evoked a ‘gut-feeling’ for which children were particularly ill, and eight participants (the most experienced clinicians) could recall examples where an intuitive response had been triggered, often in the presence of normal clinical parameters, which had led to immediate intervention.

‘It is those ones that you have a bad feeling about—those are generally the sicker ones’.

[Senior PEM clinician 010]

‘There weren't any critical signs. It wasn't her sats because she was fine. It was the fact that she didn't get up and play even with a normal temperature’.

[Clinical Trainee Paediatrics 014]

The extent to which these three supplementary skills were applied varied according to the clinician's experience, with the observation of patients' appearance being reported more frequently by less-experienced trainees as a primary factor in their decision making. Observation of behavioural clues and intuition were features described by more experienced doctors and nurses in relation to their approach to decision making.

### The transition to clinical expert

Experience proved to be (1) the vehicle to learning in this environment, (2) the pathway to development of intuition and ultimately (3) elevation to expert status. Experience developed as clinicians were exposed to a large volume and variety of clinical cases, allowing them to contrast many situations and levels of severity. This clinical experience went beyond gaining of basic competencies, and other important supplementary knowledge was developed that supported clinicians in becoming autonomous decision makers.

The process of clinicians' development of supplementary skills to aid decision making in the PED was achieved in four stages:

#### Clinical knowledge

Clinicians reported that when they were new to the PED, they felt equipped with solid clinical knowledge but little in the way of experience. Analytical-based decision making was supported by the use of guidelines and awareness of clinical features in the absence of clinical experience.

‘I think you are very aware that you might have some theoretical knowledge, but you don't have much experience’.

[Clinical Trainee Emergency Medicine 008]

‘Guidelines really, it's always nice to follow them. It's nice to be reassured that you're probably doing something that is evidence based. I think what I practice is generally from guidelines I have seen’.

[Clinical Trainee PEM 003]

#### Seeking support from colleagues

Participants described the advantage of the PED clinical team containing senior colleagues with whom less-experienced clinicians could confer in cases of uncertainty. Availability and approachability of senior and/or permanent colleagues was crucial in making management decisions in situations not previously encountered.

Part of the advantage of proximity of senior staff was also about the *type* of information gained by less-experienced staff. Acquisition of tacit knowledge contributed to the learning journey and was thought to be a result of interaction with colleagues. This was less well defined by participants within this study (four of whom raised the issue directly) but was felt to be related to the way experienced staff approached situations, and their knowledge of the department and processes. Discussion around this topic strongly suggested the importance of ‘team’ working environment as described below.

‘We have the added advantage of they [children] will come in and the triage nurse will already have seen them. She will set the ball rolling with, ‘The observations are fine’. They’re not actually too bad at the moment’.

[PEM Consultant 001]

‘Yes, but then, I have got other people in the background that I can ask. It is a good place to be’.

[Clinical Trainee Emergency Medicine 008]

#### Risk tolerance

Several participants mentioned ‘risk management’ when describing their approach to clinical decisions. Others alluded to risk tolerance/management less explicitly, but it was clear from all accounts that each clinician making a decision about the care of a child was balancing ‘risk’ and ‘safety’, particularly when making decisions regarding admission or discharge; in many situations, clinicians described children being admitted until the odds of safety could be increased.

‘The safest thing from a risk management perspective is to admit someone, isn't it, because you can always discharge them?’ [Clinical Trainee GP 007]

A culture to admit if in doubt was a feature used by trainees. Nursing staff also raised issues relating to ‘risk’ drawn, for example, from their experience of triage. All PED staff types therefore applied skills of risk management to clinical situations to varying degrees.

The acceptance of risk, the discomfort it generated and the management of potentially high-risk clinical situations were spontaneously reported during 10 interviews.

‘[In paediatrics] you want to make ‘safe’ decisions and not ‘probably fine’ decisions’.

[Clinical Trainee GP 006]

‘The stakes are so high. The parents are adding another element and I just don't see that many children’.

[Clinical Trainee several weeks into PEM rotation 008]

Clinicians from non-paediatric backgrounds reported apprehension around the ‘risk’ presented by unwell young children. Risk tolerance was crucial in many situations and was a specific skill refined through increased experience in a number and variety of clinical scenarios with support from the wider team, a key contributing factor. Under these conditions, clinicians were able to develop their own tacit knowledge permitting them to test the parameters of risk, moving away from analytical decision making and moving towards intuitive thinking. In contrast to the discomfort described by less-experienced clinicians, PEM subspecialty staff revealed their adaption to risk tolerance.

‘When you start off you want them [patients] to be perfect before you let them go home’.

[PEM Clinical Trainee 003]

‘I think there comes a point where you realise that you can comfortably handle a degree of risk, appropriately, but I think that does take a while’.

[PEM Consultant 001]

#### Intuition

The final stage of the cycle is emergence of intuition and confidence in clinical judgement to support effective decision making. Application of intuition was observed in those who made clinical decisions autonomously. It was suggested by five participants that clinicians in the PED developed intuition quicker than other environments (eg, emergency medicine) owing to the exclusive subgroup of patients.

‘As you get more experienced, then, your gut gets better at selecting out the child who is unwell’.

[PEM consultant 010]

‘Maybe because we only see Children…it means we are faster when deciding which ones are sicker’.

[ENP 013]

‘Sometimes, you can't put your finger on it. You see a child and go, ‘I am not quite happy.’ You cannot say anything more than that’.

[Band 6 Nurse 015]

## Discussion

This is the first study to demonstrate PED staff approaches to clinical decision making in young children with minor respiratory illness. Our results identify the key skills used to supplement biomedical indicators in order to achieve treatment decisions in this setting.

Application of supplementary skills was key to rapid decision making and was developed by staff on their transition to clinical expert, a journey which emerged as a strong theme, and discussed by those interviewed. Clinicians initially relied on good clinical knowledge and awareness of guidelines as a foundation to practice. This was supplemented by accumulation of experience; the exposure to clinical situations allowing clinicians to experiment with risk, eventually giving rise to the development of intuition. The role of postgraduate teaching was not discussed by participants, and the development of the skills described appeared to be acquired during patient interaction rather than direct teaching or theory-based learning.

Previous literature has examined the role of intuition among healthcare professionals.^19^ ^20^ In contrast to Benner's model which featured ‘knowledge’ as the lynchpin to expertise, our findings suggest that experience is the key to expert status for clinicians in the PED. In relation to ‘experience’, previous work has suggested that intuitive decision making can be achieved by individuals through a process of implicit learning that results in ‘pattern recognition’.^21^ The results from our study conform to these findings and also define how clinicians in the PED apply analytical decision making, a feature stronger in trainees who rely on evidence-based guidelines and support from colleagues while experience is being gained. This focus on guidelines by trainees and the move away from reliance on them with experience may partly explain variation in treatment approaches by senior clinicians in a number of conditions for which national guidelines exist, including childhood acute wheeze.^22^

In Van Den Bruel's study, ‘gut-feeling’ was evoked among primary care physicians by children presenting with specific symptoms in combination with parental concern. Authors suggested that this should result in a second opinion or referral.^23^ These features of intuition and clinical judgement were echoed in our study where professionals discussed the child's level of activity (particularly in relation to playing) as a strong indicator of possible serious illness and a factor that prompted further action by the clinician.

This study adds to the literature supporting the idea that intuition is important in clinical decision making and attention should be given to its development in training. However, clinicians should not rely solely on intuition as a decision-making aid to avoid the possibility of slipping in to complacency, or justify overtreatment. Instead, the development of intuition described here refers to its correct application to support quick, effective management decisions, but questions remain regarding whether it has the potential to increase safety or reduce paediatric admissions. The findings of our study have provided the foundation for further work in this area to be tested. The level of consistency we identified in the accounts between nursing staff, ENPs and doctors was previously undescribed, with accounts of developing and applying skills of clinical intuition very similar between groups. This may reflect the shared experiences of the staff in this environment, potentially supporting the transferability of these findings into other PEM settings.

Staying with PEM, Geelhoed's research examined the impact of increasing the number of emergency consultants in the PED. The presence of additional senior staff facilitated extra training for juniors, greater patient satisfaction and a 27% reduction in admissions.^24^ In our study, the availability of senior staff (both nurses and doctors) was cited by participants as positive in their learning experience and contributed to the development of the supplementary skills described.

It may, therefore, indicate that contact time with senior staff and the continual gain of clinical experience provide the ideal environment for junior clinicians to learn, which could have advantageous implications for delivery of patient care in PEM.

The identification of supplementary clinical skills in experienced PEM clinicians and the pathway to development of these raises the possibilities to enhance training for Primary Care Practitioners in efforts to manage PCSCs in the community and to support reduction in urgent care demand. It would be beneficial to test the development and application of these skills more extensively among other groups of professionals to assess how much experience is sufficient to achieve expert status and to evaluate this against admission reduction.

### Limitations

The study was conducted in a single site PED of a specialist children's hospital in the UK and involved a small number of participants. The findings may differ in other disciplines of medicine (particularly those involving adult patients) and in other PED settings. However, we achieved data saturation in this setting, where no new themes emerged from the participant interviews.^14^ ^17^ Transferability of the findings may be more likely in PEM where the experiences described here are recognisable to clinicians specialising in this area. The host PED was typical in terms of annual census,^25^ and there is no reason to believe that clinician opinions would vary drastically from other PEM professionals owing to the consistency in accounts. Greater volumes of clinician accounts would be beneficial to test the integrity of the themes we have identified, but no other evidence with regard to clinical decision making for young children could be located.

Ethical approval was sought to observe and interview parents attending the PED using methods of ethnography, but was declined owing to the perceived distress interviews might cause families in emergency circumstances. The lack of parent representation is a missing facet of this study. We anticipate that demand on urgent care is generated by both attendances and admissions and in order to address the issue of ED use for PCSCs, patient factors responsible for initiating requests for urgent care must also be fully considered.

Similarly, we have not explored the impact of external factors on clinical decision making for young patients, or issues affecting service delivery such as the role of Paediatric Assessment Units, clinician accountability and ED quality indicators^26^ (including the 4-hour target).

The impact of these and of families' experiences on ED use is primed for further research to identify and assess the potential for both education strategies for clinicians and support mechanisms directed at families to continue to address the issue of high urgent care demand.

## Conclusion

When faced with young children in the PED with cases of minor respiratory illness, clinicians are required to assess and achieve rapid decisions, balancing aspects of patient safety and use of resources. We have identified the key supplementary skills of observation, risk tolerance and intuition gained through extensive clinical experience.

Having identified these skills in the PED that enhance decision making, and described how they are acquired, we suggest that learning programmes could be developed and used in combination with other strategies to address the growing demand on paediatric urgent care.
