# Supplementary material for: The transition to clinical expert: enhanced decision making for children aged less than 5 years attending the paediatric ED with acute respiratory conditions
Source: Emerg Med J. 2016 Aug 5;34(2):76–81. doi: 10.1136/emermed-2015-205211 (PMC5384604; doi:10.1136/emermed-2015-205211)
Supplement: Supplementary data [file emermed-2015-205211supp001.pdf]

Section 1. Pre interview

- Briefly review aim of study and interview, opportunity for questions, confirm that interview will take no longer than forty minutes and will be recorded, take written consent.

Participant background

- Background information on participant e.g. job title, length of time qualified/working at the PED

Section 2. Management of minor respiratory illness in the PED (general)

- What does the term 'minor respiratory illness' mean to you? What conditions do you believe this description would include?
- In your experience, how often do cases of minor respiratory illness present to the PED? Are there spikes/peaks and if yes, when?
- How are minor respiratory illnesses managed in the PED? Describe a typical treatment path.
- Could you think of/describe a typical clinical situation where a child presents but they could be managed more appropriately in the community?
- Can you tell me about cases where community management would not be clinically appropriate.
- Could you tell me about possible 'grey area' cases that first appear to be minor but really need to be treated?

Section 3. Clinician decision making in real-life clinical cases

When I contacted you about an interview, I asked if you could think about two real examples of clinical cases involving young children who have presented to the PED with minor respiratory illness.

For each case:

- Can you tell a bit about the case, how they presented, symptoms, background
- How ill did the child appear to you on first encounter? How concerned were you at this point and what about? To what extent was there anything typical/unusual about this case?
- What factors, if any, complicated the presentation? (Prompt for example for pre-existing illness, parental concern, unusual symptoms, previous visits to other HCPs)
- At what point did you decide how this child should be treated?
- How did you make this decision? What things influenced your decision making and why?
- Describe what course of action was taken.
- Retrospectively, would you take this management approach again? Why or why not?

Section 4.Knowledge of and communication with families

- Why do you think parents present for minor respiratory problems? In what situations do you think it's appropriate/inappropriate?
- In your experience, are some parents more likely to come to PED? What factors influence parents to come?

- In your view, what knowledge do parents have of minor respiratory illness and how/where to seek help?
- What do you seek to communicate to parents in consultations for minor respiratory illness? (Prompt for example for advice about other services, how to manage at home, when to seek further help, how to manage this situation in the future). How is this received by parents/carers?

Section 5.Interventions for appropriate management of respiratory problems

- Are there any interventions already in place in the PED to ‘filter’ minor illnesses (or in existence that you’re aware of)? What are these and how effective do you believe these are?
- What would be your ideal intervention for improving the management of non-life-threatening respiratory problems?
- What would be the barriers and enablers for such an intervention to be implemented?

Close

- Anything else?
- Thank participant and ask if they would like a copy of their transcript
- Describe how findings will be fed back
